# Evolutionary Adaptation of an RNA Bacteriophage to Repeated Freezing and Thawing Cycles

**DOI:** 10.3390/ijms25094863

**Published:** 2024-04-29

**Authors:** Mara Laguna-Castro, Alicia Rodríguez-Moreno, Ester Lázaro

**Affiliations:** Centro de Astrobiología (CAB), CSIC-INTA, Carretera de Ajalvir Km 4, 28850 Torrejón de Ardoz, Madrid, Spain; mlaguna@cab.inta-csic.es (M.L.-C.); arodriguez@cab.inta-csic.es (A.R.-M.)

**Keywords:** bacteriophage Qβ, evolutionary optimization, experimental evolution, freezing-thawing stress, adaptation, extracellular selective pressures

## Abstract

Bacteriophage fitness is determined by factors influencing both their replication within bacteria and their ability to maintain infectivity between infections. The latter becomes particularly crucial under adverse environmental conditions or when host density is low. In such scenarios, the damage experienced by viral particles could lead to the loss of infectivity, which might be mitigated if the virus undergoes evolutionary optimization through replication. In this study, we conducted an evolution experiment involving bacteriophage Qβ, wherein it underwent 30 serial transfers, each involving a cycle of freezing and thawing followed by replication of the surviving viruses. Our findings show that Qβ was capable of enhancing its resistance to this selective pressure through various adaptive pathways that did not impair the virus replicative capacity. Notably, these adaptations predominantly involved mutations located within genes encoding capsid proteins. The adapted populations exhibited higher resistance levels than individual viruses isolated from them, and the latter surpassed those observed in single mutants generated via site-directed mutagenesis. This suggests potential interactions among mutants and mutations. In conclusion, our study highlights the significant role of extracellular selective pressures in driving the evolution of phages, influencing both the genetic composition of their populations and their phenotypic properties.

## 1. Introduction

Bacteriophages, or just phages, are the most abundant biological entities in the biosphere, outnumbering their bacterial hosts by at least a factor of 10 [1]. Current estimates suggest that the number of phages on our planet may reach as high as 10^31^ [2]. Although, due to their small size, they constitute only a small fraction of the total biomass, their ecological impact is highly significant. Firstly, the high number of phage infections that occur daily stands as one of the most relevant factors in regulating the structure and composition of bacterial communities, not only in aquatic or terrestrial ecosystems [3,4,5], but also within organisms [6,7]. Additionally, the lysis of infected bacteria results in the release into the environment of dissolved organic matter (viral shunt) rich in nucleotides and amino acids that can be consumed by the lower levels of the trophic chains, helping nutrient recycling [8,9] and contributing to the regulation of biogeochemical cycles of elements such as carbon, sulfur, iron and phosphorus [10,11,12,13,14]. Finally, it should also be pointed out that bacteriophages are one of the most important evolutionary drivers in the bacterial world, acting not only as a selective pressure that favors the emergence of resistance mechanisms [15,16], but also as agents of horizontal gene transfer and as generators of genetic diversity [17,18,19,20].

The life cycle of lytic bacteriophages comprises two distinct phases: an extracellular phase, during which they remain as inert particles, and an intracellular phase, wherein they actively replicate within infected bacteria. In environments with high host densities, phages readily initiate infections, so their evolution is mainly driven by intracellular selective pressures that affect their capacity to generate an infectious progeny. However, the dynamics of bacteria–phage relationships, often described as a predator–prey system [21,22,23], can lead to periods in which bacteria susceptible to infection are scarce. When that happens, phages must stay longer in the extracellular environment, where they are vulnerable to the detrimental effects of external physicochemical agents, potentially damaging viral particles and rendering them non-infectious. This kind of population bottlenecks driven by external agents may have a strong influence on subsequent phage evolution. It is easy to understand that phages exhibiting greater resilience to environmental stresses are more likely to initiate new infections when hosts become abundant once again. Thus, it is foreseeable that populations will become enriched in those variants, even if they are not the ones that better replicate within the cell [24,25,26]. In this context, a study involving 16 phages infecting *Escherichia coli* suggested a conflict between optimizing persistence in the environment and replication within the host [27]. Similarly, evolution experiments carried out with vesicular stomatitis virus, propagated in vitro under conditions that prolonged the time the virus spent outside the host, revealed the selection of viruses capable of surviving longer in the environment, albeit at the expense of producing fewer progeny [28,29]. Conversely, other studies involving bacteriophages exposed to low pH or increased temperature in the periods between replications did not demonstrate a correlation between enhanced resistance to adverse extracellular conditions and diminished replicative capacity [30,31].

There are many environmental variables that can perturb the structure of viral particles, such as high temperatures, ultraviolet radiation, extreme pH levels, or high salinity. Remarkably, many microorganisms thrive in environments where such conditions are the norm, each hosting characteristic viruses that have evolved strategies to persist stably in that inhospitable surrounding [32,33,34]. The study of these strategies, as well as those employed by mesophilic viruses to adapt to adverse circumstances is of great interest in evolutionary biology.

Using the bacteriophage Qβ as the experimental model, we have established a serial transfer protocol that allows us to study the evolution of this virus under controlled conditions in the laboratory. This phage belongs to the *Fiersviridae* family (formerly named *Leviviridae*), genus Qubevirus [35]. It infects the bacterium *E. coli* using the conjugative F pilus as receptor. Its genome is a single-stranded, positive-sense RNA chain, which is 4217 nucleotides long and encodes for four proteins: the A2 protein, necessary for cell receptor recognition, penetration of the phage genome and lysis of the infected bacteria [36,37]; the coat protein, which is the major capsid protein; the A1 protein, produced by misreading of the stop codon of the coat protein and present in low proportion in the capsid [38]; and the replicase [39], necessary to copy the RNA genome. Similar to other RNA viruses, bacteriophage Qβ replicates with high error rates and forms highly heterogeneous populations [40], making it able to rapidly adapt to a wide variety of environmental conditions. In the last years, we have conducted a number of studies concerning the adaptation of this virus to diverse selective pressures, including increases in the intracellular temperature, exposure to extracellular heat shocks, and the combined influence of both [41,42,43,44]. The results obtained showed that adaptation to extracellular heat shocks occurred through a single mutation in the A1 protein of the viral capsid [31]. Conversely, adaptation to temperature increases during replication took place through a set of mutations distributed across all virus genes [41,42,43,44]. Simultaneous adaptation to both conditions resulted in the selection of the same mutations that were selected when each selective pressure was applied separately [31].

In order to delve deeper into how the environmental conditions prevailing when phages are not replicating influence their evolution, this study explores whether bacteriophage Qβ is able to adapt to a selective pressure that involves freezing and thawing of viral particles. Qβ infects *E. coli* in the human gut, from where it can be excreted into the environment and transmitted via the fecal–oral route. Hence, exposure to freezing and thawing cycles presents a challenge not typically encountered by this virus. Understanding the impact of exposure to this condition, either alone or alternating with replication cycles inside the cell, may shed light on the ability of phages to persist in ecosystems that can be highly inhospitable.

Freezing and thawing of the biological material may be relevant in environments, such as Arctic permafrost, tundra regions or Antarctic lakes, where temperatures may drop below the freezing point of water and then rise sufficiently to allow thawing to occur. Under these circumstances, ice crystals form that can damage biological molecules and structures and, in the case of viruses, lead to the shedding of genetic material [45,46]. Proteins that interact with the cell receptor might also be damaged and, thus, they constitute another potential target for evolutionary optimization.

The evolution experiments performed with bacteriophage Qβ show that this virus can increase its resistance to exposure to freezing and thawing cycles without affecting its replicative capacity. The adaptive pathways followed are diverse and involve the acquisition of mutations preferentially located in the capsid proteins. Taken together, our results illustrate that the ability of phages to retain their infectivity in the extracellular environment is a fundamental fitness trait that is subject to evolutionary optimization.

## 2. Materials and Methods

### 2.1. Viruses and Bacteria

The plasmid pBRT7Qβ, which contains a cDNA of bacteriophage Qβ cloned in the plasmid pBR322 [47,48], was a generous gift from Professor CK Biebricher (Max Planck Institute for Biophysical Chemistry, Göttingen, Germany). It was used to transform *Escherichia coli* DH5-α (MAX Efficiency^TM^, Invitrogen, Waltham, MA, USA), a strain that permits virus expression, although it cannot be infected because it lacks the virus receptor. The supernatant of an overnight culture, obtained from a transformed colony, was used to infect *E. coli*, strain Hfr (Hayes) [49], in semisolid agar. The virus progeny contained in a randomly chosen lysis plaque was isolated, and 10^6^ plaque forming units (pfu) were used to infect an *E. coli* Hfr culture under standard conditions (37 °C, 250 rpm) for 2 h. The supernatant of this culture was used as the ancestor of all the evolutionary lines analyzed in this work. It was denoted Qβ_Anc_ and its consensus sequence showed no mutations relative to the Qβ cDNA cloned in pBR322.

### 2.2. Replication Assays in Liquid Medium

Standard infections in liquid medium were carried out using 0.5 mL of fresh log-phase *E. coli* cultures (OD_600_ of ~0.8) that were infected with the amount of pfu indicated in each experiment in a volume of 100 µL of phage buffer (25 mM Tris HCl pH 7.5, 5 mM MgCl_2_, 1 g/L gelatin from Merck, Darmstadt, Germany). The final volume was completed to 1 mL with NB medium (8 g/L Nutrient Broth from Merck and 5 g/L NaCl). Cultures were incubated at 37 °C for 2 h with good aeration (250 rpm) in a New Brunswick Scientific Innova 42 Incubator Shaker (Eppendorf, Enfield, CT, USA). To estimate the total virus yield, cultures were treated with chloroform (1/20 *v*/*v*, 28 °C, 15 min, shaking 850 rpm in thermoblock). Virus supernatants were harvested upon centrifugation at 13,000× *g* and maintained at 4 °C for short-term use (less than 15 days) or at −80 °C for long-term storage. When necessary, virus supernatants were diluted in phage buffer. Virus titers were determined by plaque assay and expressed as the number of pfu per mL of the phage suspension.

### 2.3. Evolution Experiment

**Figure 1:**
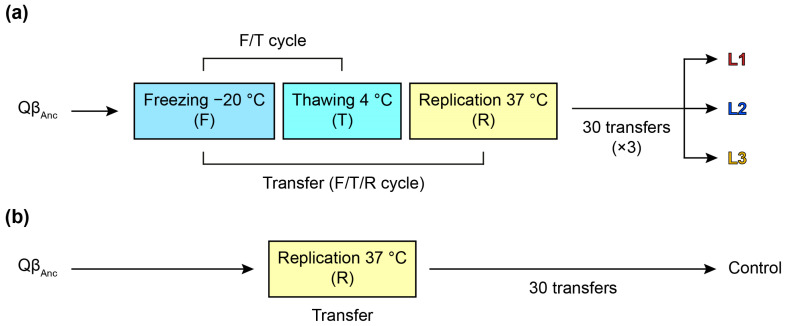
Scheme showing the evolution experiment performed with bacteriophage Qβ. (**a**) The virus Qβ_Anc_ was propagated in triplicate through 30 serial transfers consisting of freezing at −20 °C, thawing at 4 °C, and amplification of the surviving viruses in a standard replication assay. The lines obtained were denoted L1, L2 and L3 (evolved lines). (**b**) The virus Qβ_Anc_ was propagated through 30 serial transfers that did not include freezing and thawing. In this way, a control line was obtained whose properties were compared with those of the ones evolved through F/T/R cycles.

The virus Qβ_Anc_ was used to initiate 3 replicate evolutionary lineages (L1, L2 and L3) that were propagated for 30 serial transfers (Figure 1). Each transfer consisted of a cycle of virus freezing and thawing (F/T), followed by the replication (R) of a fraction of the surviving viruses in liquid medium. At all transfers, except the first that was initiated with 7 × 10^8^ pfu, the amount of virus that was frozen at −20 °C was 10^8^ pfu in a volume of 1 mL of phage buffer. The sample was kept at −20 °C for 16–18 h, then thawed at 4 °C and titrated. An aliquot (100 µL) of the thawed sample was replicated in *E. coli* under standard conditions (2 h, 37 °C, 250 rpm). The supernatant of the culture was titrated upon treatment with chloroform, and diluted to a concentration of 10^8^ pfu/mL to initiate a new transfer (a complete F/T/R cycle). The process was repeated for 30 F/T/R cycles.

In all transfers, a record was kept at −80 °C of both the samples obtained after the F/T cycles and those obtained after virus replication. A control line was also established in which the ancestral virus was subjected to 30 transfers consisting only of replication cycles, without intermediate freezing and thawing. In this case, the amount of virus used to initiate each transfer was 10^7^ pfu of the previous supernatant.

### 2.4. Resistance to Freezing and Thawing

To assess the resistance of virus lines and clones to freezing and thawing, they were subjected to three consecutive F/T cycles. Initially, 1 mL of phage buffer containing a specified quantity of virus was frozen at −20 °C for ~16 h and then thawed at 4 °C. The surviving viruses were quantified by titrating an aliquot (10 µL) of the sample, and the rest of it was frozen again. The process was repeated until completing the three F/T cycles. The survival rate was calculated as the quotient between the virus titer after the third cycle and the virus titer at the onset of the experiment. In order to compare the values obtained in different assays, the survival rate of the viruses under analysis was divided by that obtained for the ancestral virus in the same assay, which was used as an internal control for each experiment. In this way, we obtained normalized survival rate values.

### 2.5. Determination of the Replicative Capacity

The virus yield obtained in standard replication assays was used as a measure of the virus replicative capacity. Accordingly, triplicate liquid cultures, prepared as described in the Section 2.2, were inoculated with 10^4^ pfu of the virus population assayed, in a final volume of 1 mL. In previous experiments we had shown that, under the conditions assayed, 10^4^ pfu is within the range in which the progeny produced grows exponentially as a function of the initial amount of virus used to infect.

### 2.6. Site-Directed Mutagenesis

The plasmid pBRT7Qβ was used to engineer the single-mutant viruses Qβ_G1312A_, Qβ_G1494A_, Qβ_U1665C_, Qβ_A1930G_, Qβ_C2001U_ and Qβ_G2223A_, which contain the mutations indicated as subscripts. Mutagenesis was carried out using the QuickChange Lightning Site-Directed Mutagenesis Kit (Agilent Technologies, Santa Clara, CA, USA) with the primers shown in Table S1 that carry the mutations to be introduced into the virus genome. The procedures to build and isolate the site-directed mutants were the same as described previously for other Qβ mutants [50]. A lytic plaque generated in *E. coli* Hfr by each of the mutants was picked and sequenced to test the presence of the desired mutation and the absence of any others that might have arisen during the mutagenesis process.

### 2.7. Isolation of Biological Clones

Biological clones corresponded to lytic plaques obtained in semisolid agar in *E. coli* Hfr grown at 37 °C upon plating the evolved lines (L1, L2 and L3) or the virus Qβ_Anc_. They were isolated by punching and removing the top and the bottom agar around well-separated lytic plaques as previously described [51,52]. The agar containing each plaque was transferred into a tube with 1 mL of phage buffer and 50 μL of chloroform, and incubated for 1 h at 25 °C with shaking (900 rpm). After centrifugation at 13,000× *g* for 15 min to clarify the supernatant, the latter was stored over 25 μL of chloroform. Clones isolated from the evolved lines were named LxCy, where x designates the line from which they were isolated and y is a number that was arbitrarily assigned. Clones isolated from the virus Qβ_Anc_ were denoted Qβ_Anc_(1), Qβ_Anc_(2) and Qβ_Anc_(3).

### 2.8. RNA Extraction, cDNA Synthesis, PCR Amplification and Nucleotide Sequencing

Viral RNA was prepared following standard procedures to determine the consensus sequence either from biological clones or from complex virus populations. RNAs were used for cDNA synthesis with the avian myeloblastosis virus reverse transcriptase (Promega), followed by PCR amplification using Expand high-fidelity DNA polymerase (Roche). The pairs of oligonucleotide primers used for RT-PCR were the following: P1 forward (5′CTTTAGGGGGTCACCTCACAC3′) with P1 reverse (5′GGATGGGTCACAAGAACCGT3′) to amplify from nucleotide position 10 to 1595, P2 forward (5′GACGTGACATCCGGCTCAAA3′) with P2 reverse (5′CAACGGACGGAACATCTCCT3′) to amplify from nucleotide position 1109 to 2787, and P3 forward (5′GTGCCATACCGTTTGACT3′) with P3 reverse (5′GATCCCCCTCTCACTCGT3′) to amplify from nucleotide position 2254 to 4195. PCR products were column purified (Qiagen, Hilden, Germany) and subjected to standard Sanger sequencing using Big Dye Chemistry (v3.1) with an automated sequencer (Abi 3730 XL, Applied Biosystems, Perkin Elmer, Waltham, MA, USA). Sequences were assembled and aligned with Geneious Pro v4.8.5 (https://www.geneious.com). Mutations relative to the sequence of the Qβ cDNA present in the plasmid pBRT7Qβ (virus Qβ_Anc_) were identified using the same software.

### 2.9. Statistics

Statistical analyses were performed with R (version 4.3.2). All analyses were two-tailed and statistical significance was set at *p* < 0.05. Statistical differences were assessed using one-way ANOVA followed by post-hoc Dunnett’s multiple comparison test.

All experiments were performed with three biological replicates with the exception of the analysis of the resistance to freezing and thawing of biological clones isolated from evolved populations, which was performed in duplicate. The degree of statistical significance is indicated in the text with asterisks: (*) *p* < 0.05, (**) *p* < 0.01 and (***) *p* < 0.001.

## 3. Results

### 3.1. Sensitivity of Bacteriophage Qβ to Freezing at −20 °C Followed by Thawing at 4 °C

**Figure 2:**
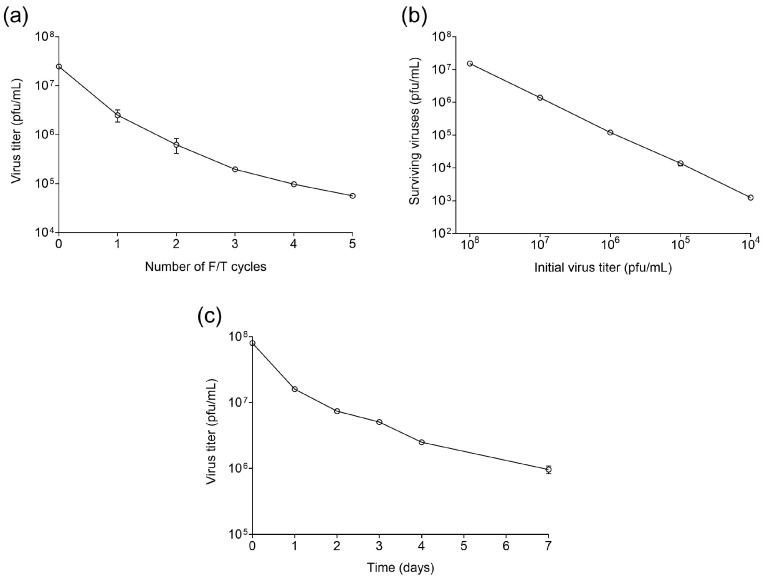
(**a**) Titer decay experienced by the virus Qβ_Anc_ when subjected to 5 consecutive F/T cycles. (**b**) Number of surviving viruses (pfu/mL) after an F/T cycle as a function of the initial concentration of the virus Qβ_Anc_. (**c**) Titer decay experienced by the virus Qβ_Anc_ after an F/T cycle as a function of the time that the virus remained frozen at −20 °C. All determinations were carried out in triplicate. The error bars represent the standard deviation of the three replicas. When error bars are absent it is because they were too small to be observable.

To test the sensitivity of bacteriophage Qβ to freezing (−20 °C) and thawing (4 °C), we prepared two samples of 1 mL containing 2.5 × 10^7^ pfu/mL of the virus Qβ_Anc_. Both replicas were frozen at −20 °C and kept in the freezer for ~18 h. After this time, they were thawed at 4 °C, titrated (for which a 10 µL aliquot was taken and diluted appropriately), and kept at this temperature for ~6 h. The remaining volume was frozen and thawed again using the same conditions as described above. The procedure was repeated for five F/T cycles. We observed (Figure 2a) that the virus titer decreased progressively each time it was frozen and thawed.

To analyze the impact of the phage concentration on its sensitivity to freezing and thawing, a sample of the virus Qβ_Anc_ was serially diluted four times. Samples containing 1 mL of each dilution were frozen at −20 °C for 18 h, thawed at 4 °C and titrated. Remarkably, all dilutions exhibited a proportional decrease in their titers (approximately fivefold, which represents a survival rate of 0.2), suggesting that virus concentration does not significantly impact the extent of damage incurred when Qβ is subjected to freezing at −20 °C followed by thawing at 4 °C (Figure 2b).

Then, we determined whether the time that the viral particles remained at −20 °C had some influence in the virus titers recovered after thawing. To do that, we simultaneously froze five Qβ_Anc_ aliquots (each containing 8 × 10^7^ pfu/mL in a volume of 1 mL). The samples were thawed at 4 °C at different intervals, so that the first sample remained frozen for 24 h and the last one for 168 h. Titration of the thawed samples showed that the longer the virus remained at −20 °C the greater the decrease in their titers (Figure 2c). The most pronounced drop occurred in the sample that was thawed first, suggesting that is the freezing–thawing process that produces the greatest damage to the virus, although this can be increased if the time the virus remains at −20 °C is longer.

Finally, we performed an assay in which we determined whether the replicative capacity of Qβ was affected after an F/T cycle. For this purpose, we performed a replication assay in liquid medium, using 10^7^ pfu of two Qβ_Anc_ samples—one that had not been frozen at −20 °C and another one that had been subjected to an F/T cycle. Both samples were tested in triplicate. The titers (mean ± standard deviation) obtained after two hours of replication at 37 °C under standard conditions were quite similar: 7.0 × 10^10^ ± 1.2 × 10^10^ pfu/mL and 7.5 × 10^10^ ± 1.6 × 10^10^ pfu/mL, respectively, indicating that the viruses that survive after an F/T cycle do not reduce their replicative capacity under the conditions assayed.

### 3.2. Evolution Experiment and Characterization of the Evolved Populations

In order to study whether Qβ could increase its resistance to the exposure to F/T cycles, we designed an evolution experiment in which the virus Qβ_Anc_ was propagated through 30 serial transfers, each consisting of an F/T cycle, followed by the amplification of a fraction of the survival viruses in a replication assay carried out under standard conditions (see Figure 1 and Section 2). The design of the experiment means that the virus evolves in the face of two different selective pressures that will favor viruses that better resist freezing and thawing and are also able to replicate. A control line was also propagated through 30 serial transfers that involved only the step of virus replication, without intermediate F/T cycles in between (see Figure 1 and Section 2).

**Figure 3:**
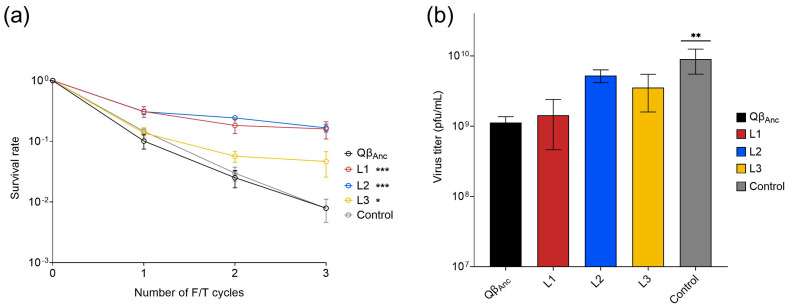
Survival rate and replicative capacity of the three Qβ evolved lines (L1, L2 and L3), the control line, and the virus Qβ_Anc_. (**a**) Survival rate. Each line was subjected to three consecutive F/T cycles. The survival rate was calculated with respect to the initial amount of virus (~10^7^ pfu in a volume of 1 mL). The error bars represent the standard deviation of the three replicas assayed. The asterisks indicate the statistical significance of the value obtained for each line in cycle 3 with respect to that of the virus Qβ_Anc_ (see Section 2.9). (**b**) Replicative capacity_._ The assay was performed as indicated in Section 2. The error bars represent the standard deviation of the three replicas performed for each virus. Asterisks above the bars indicate the statistical significance of each line with respect to the virus Qβ_Anc_ (see Section 2.9), * *p* < 0.05, ** *p* < 0.01, and *** *p* < 0.001.

The control and the three evolved lines were assayed to determine the possible changes experienced in both the resistance to freezing and thawing and the replicative capacity. For that, each line was exposed to three F/T cycles without amplification of the surviving viruses in between. The three evolved lines kept a larger fraction of viable viruses than the ancestral virus (Figure 3a), indicating that they had increased their resistance (normalized survival rates = 19.3, 21 and 6 for L1, L2 and L3, respectively). The control line showed similar sensitivity to freezing and thawing as the ancestor, which suggests that the improved resistance in the lines evolved through F/T/R cycles is not due to the increase of the heterogeneity of the virus populations that predictably occur during the amplification steps but to the selection of particular mutations that increase the resistance.

The replicative capacity of the control and the evolved lines was assayed in a replication assay performed using a low input of virus (10^4^ pfu; see Section 2). All lines increased their replicative capacity with respect to the ancestor, although those evolved through F/T/R cycles did so to a lesser degree and the augments were not statistically significant (Figure 3b).

**Figure 4:**
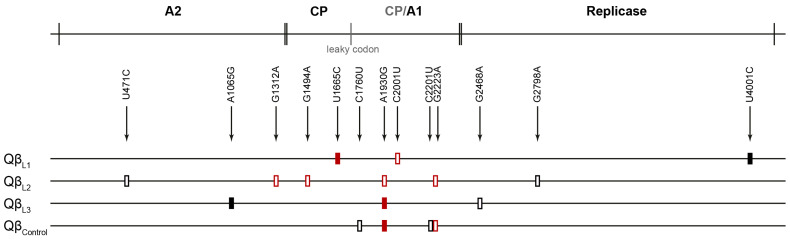
Scheme of the genome of bacteriophage Qβ showing the encoded proteins and the mutations detected in the lines analyzed. CP means coat protein. Red rectangles indicate non-synonymous mutations and black rectangles are synonymous mutations. Filled and blank rectangles refer to fixed and polymorphic mutations, respectively. See Table S2 for more detailed information.

To identify the mutations responsible for the resistance, we used Sanger sequencing to determine the consensus sequences at transfer number 30 of the control and the three lines evolved through F/T/R cycles (Figure 4 and Table S2). The control line showed four mutations that had already been detected in previous Qβ evolution experiments carried out in our lab. Regarding the mutations found in the lines evolved through F/T/R cycles, it is noteworthy that out of a total of eleven mutations, either fixed or polymorphic, only three (G1312A, A1930G and G2223A) had been previously detected in Qβ evolution experiments that did not involve extracellular selective pressures. The rest seem to be mutations exclusive of the new selective pressure (freezing and thawing) that we have applied now. Eight mutations were located in the genes encoding the capsid proteins, although two of them were synonymous. It is remarkable that, apart from A1930G, there were no matching mutations between the lines evolved through F/T/R cycles.

Looking in more detail at the non-synonymous mutations located in the capsid proteins, it is noteworthy that L1 has one mutation fixed in the coat protein (U1665U (F107L)) and another one polymorphic in the A1 protein (C2001U (L201U)); L2 has four polymorphic mutations, one located in the A2 protein (G1312A (V417I)), another one in the coat protein (G1494A (V50I)), and two in the A1 protein (A1930G (Q195R), G2223A (V293I)); and L3 only presents mutation A1930G (Q195R). Of all these mutations, G1494A, U1665U and C2001U deserve particular attention due to their absence in other Qβ evolution experiments carried out in our group. Thus, they are candidates to be responsible, at least in part, for the freezing and thawing resistance phenotype.

### 3.3. Effect of Single Mutations in the Resistance to Freezing–Thawing Cycles

We used the plasmid pBRT7Qβ to generate single mutant viruses containing each of the non-synonymous mutations identified in the capsid proteins of the lines evolved through F/T/R cycles. These mutations were G1312A (V417I) in the A2 protein; G1494A (V50I) and U1665C (F107L) in the coat protein; and A1930G (Q195R), C2001U (L219F) and G2223A (V293I) in the readthrough domain of the A1 protein.

**Figure 5:**
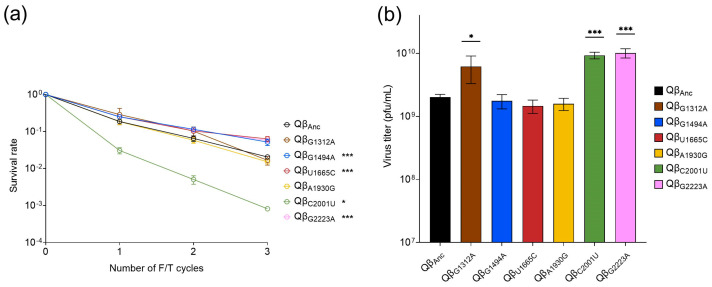
(**a**) Survival rate of the virus Qβ_Anc_ and the single mutants indicated. Each virus was subjected to three consecutive F/T cycles. The survival rate was calculated with respect to the initial amount of virus (~5 × 10^6^ pfu in a volume of 1 mL). The error bars represent the standard deviation of the three replicas assayed for each point of the experiment. The asterisks indicate the statistical significance of the value obtained for each line in cycle 3 with respect to that of the virus Qβ_Anc_ (see Section 2.9). (**b**) Replicative capacity of the virus Qβ_Anc_ and the single mutants indicated. The assay was performed as indicated in Section 2. The error bars represent the standard deviation of the three replicas performed for each virus. Asterisks above the bars indicate the statistical significance of each virus with respect to the virus Qβ_Anc_ (see Section 2.9), * *p* < 0.05 and *** *p* < 0.001.

All the single mutants were assayed to test their resistance to the exposure to three F/T cycles. The results obtained (Figure 5a) show that mutations G1494A, U1665C and G2223A increased their resistance with respect to the ancestor, although to a lesser extent than any of the evolved lines (normalized survival rate values were 2.5, 3.1 and 2.8, respectively). In contrast to this, mutations G1312A and A1930G showed lower resistance than the ancestor (normalized survival rate values of 0.8 and 0.7, respectively) although the difference was statistically non-significant. Surprisingly, C2001U showed a great sensitivity to freezing and thawing (normalized survival rate of 0.04). It is striking that a mutation that decreases resistance to freezing and thawing is maintained in a population that has experienced 30 F/T cycles. A possible explanation is that, in the resistance test, the viruses are exposed to F/T cycles without replication in between, which is an important difference with respect to the evolution experiment, where, after each exposure to an F/T cycle, the surviving viruses are amplified in a replication assay. Therefore, not only the viruses that better resist freezing–thawing are favored, but also those that replicate more efficiently. To analyze this issue, we performed a replication assay of all mutants (Figure 5b), in which it was found that those containing G1312A, C2001U, or G2223A increased their replicative capacity with respect to the virus Qβ_Anc_, while the rest of mutants presented values similar to the latter. Therefore, it seems most likely that mutation C2001U, which decreases resistance to freezing and thawing, may be maintained as a polymorphism in the evolutionary line L1 due to its positive effect on Qβ replication.

### 3.4. Resistance to F/T of Biological Clones Isolated from Evolved Populations

**Figure 6:**
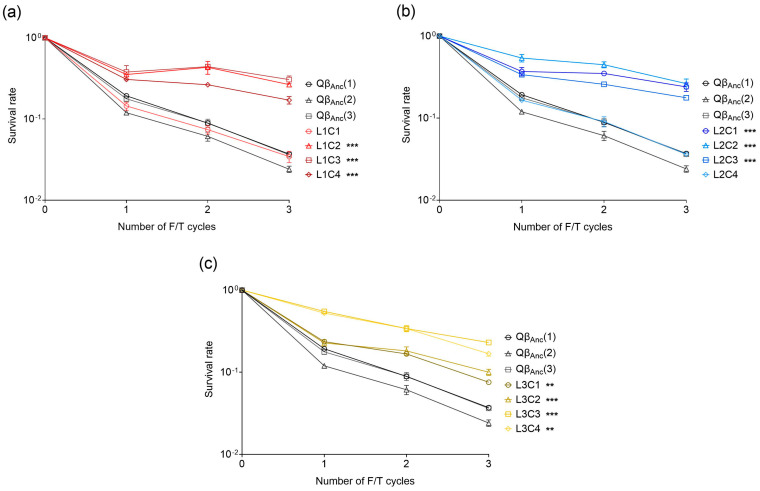
Survival rates of the biological clones isolated either from the virus Qβ_Anc_ or the lines evolved through F/T/R cycles. (**a**) Clones isolated from L1. (**b**) Clones isolated from L2. (**c**) Clones isolated from L3. In all cases the survival rate was calculated with respect to the initial amount of virus (~5 × 10^6^ pfu in a volume of 1 mL). The error bars represent the standard deviation of the two replicas assayed for each point of the experiment. The asterisks indicate the statistical significance of the value obtained for each line in cycle 3 with respect to that of the virus Qβ_Anc_ (see Section 2.9), ** *p* < 0.01, and *** *p* < 0.001.

To analyze whether different mutants isolated from a particular population differ in their resistance levels, we isolated four biological clones from each evolved line (L1, L2 and L3) and exposed them to three F/T cycles without viral replication between them. The results were compared with those obtained for three clones isolated from the virus Qβ_Anc_. Overall, virus clones coming from the evolved populations showed higher survival rates than those isolated from the ancestral population, although clones L1C1 and L2C4 did not differ significantly from the latter (Figure 6). It is remarkable that clones isolated from lines L1 and L2 have lower normalized resistance values than their respective originating lines (Table 1). However, these values were still higher than those observed in any of the single mutants tested. On the other hand, clones isolated from L3 showed normalized resistance values similar to those of the original line (Table 1).

In an attempt to establish correspondence between genetic sequences and levels of resistance, we determined the consensus sequences of the biological clones under analysis. The complete list of detected mutations can be found in the Supplementary Material (Table S3). As expected from the consensus sequences of the evolved lines, the majority of non-synonymous mutations were located in the structural proteins of the capsid (Figure 4). Additionally, there were some synonymous mutations distributed more evenly throughout the genome.

The fact that all clones displayed different combinations of mutations and varying levels of resistance makes it difficult to identify the genetic changes that are responsible for the observed phenotypic effects. As expected from the consensus sequence of L1, all clones isolated from this line carry mutations U1665C and U4001C. Virus clone L1C1 stands out with the lowest resistance level among those from L1, resembling the clones isolated from the ancestral population. Notably, this clone is the only one with mutation C2001U, which significantly impairs resistance to freezing and thawing (see Figure 5a). Interestingly, L2C4, which also exhibits resistance levels comparable to those from the ancestral population, lacks any of the mutations present in the consensus sequence of L2. It is remarkable that no clone presents resistance levels as high as those observed for lines L1 and L2 (see Table 1).

## 4. Discussion

The mechanisms used by viruses to persist in the environment under conditions that diminish their infectivity have received much less attention than those optimizing their replication within the cells. Nonetheless, it is crucial to recognize the significance of both sets of mechanisms, since if a virus is very sensitive to the damage caused by external physicochemical agents, it may become extinct, even if its replicative capacity is high. This becomes particularly pertinent in scenarios where hosts are scarce, highlighting the importance of maintaining infectivity between replication cycles. There are numerous examples of viruses that regularly endure adverse environmental conditions [32,33,34], which drive the evolution of adaptive mechanisms to withstand them. In addition to characterizing the viruses naturally thriving in such environments, experimental evolutionary studies carried out under controlled conditions in the laboratory offer another valuable approach to understand the molecular mechanisms of adaptation to specific selective pressures [53,54,55,56,57,58]. Our study investigates whether bacteriophage Qβ can adapt to an extracellular selective pressure that is not common for it: freezing and thawing of viral particles. By examining the impact of this condition on phage survival, either alone or in conjunction with replication cycles, we aim to elucidate how harsh extracellular selective pressures shape evolutionary trajectories of phages at both phenotypic and genetic levels.

Our initial experiments revealed that, after five F/T cycles, Qβ titers were reduced by more than three orders of magnitude (Figure 2a), suggesting that, if the process had continued, the virus would have been extinguished. Notably, the fraction of virus that lost infectivity was independent of the concentration of viral particles and not directly proportional to freezing duration (Figure 2b,c). Despite these adverse effects on Qβ infectivity, we anticipated that productive infections between cycles could mitigate the damaging impact. During replication, amplification of mutants with improved resistance to freezing and thawing might occur, while new mutants with adaptive potential could also be generated. In our evolution experiment (Figure 1), we simulated this scenario, allowing Qβ replication under optimal conditions after each F/T cycle. Under these circumstances, the virus faced selective pressures favoring variants resistant to freezing and thawing while maintaining replicative efficiency. Although initially, viruses surviving F/T cycles replicated similarly to those not exposed to this condition, subsequent enrichment in specific mutants could alter replication dynamics.

Our findings revealed a notable increase in survival rates following three consecutive F/T cycles in the three evolved lines, a phenomenon not observed in the control line. Furthermore, the evolved lines exhibited an enhanced replicative capacity compared to the ancestral virus, albeit to a lesser extent than the control line that remained unexposed to F/T cycles. Genetic analysis (Figure 4 and Table S2) showed that the control line had acquired several mutations frequently associated with Qβ evolution in the absence of extracellular stresses. Remarkably, the lines evolved through F/T/R cycles also acquired some of these mutations (G1312A (L2), A1930G (L2 and L3) and G2223A (L3)), which might explain the observed increases in their replicative capacity. In addition, these lines harbored novel mutations likely responsible for the augmented survival rates after freezing and thawing (U471C, A1065G, G1494A, U1665C, C2001U, G2468A and G2798A). Notably, non-synonymous mutations in the coat protein (U1665C in L1 and G1494A in L2) are of particular significance, as they induce amino acid changes repeated in all capsid subunits, potentially increasing resistance. Mutation C2001U, also non-synonymous, located in the A1 protein—essential for infectivity [38] and potentially serving as primary binding sites for cellular receptors [59,60]—adds further complexity to the adaptation process. The remaining mutations included in the above list are synonymous and affect all genes of the virus. Thus, the enhanced resistance to freezing and thawing seems to be due to a combination of non-synonymous mutations altering capsid structure to make it more resistant and synonymous mutations likely influencing viral RNA structure, thereby modifying internal pressure exerted within the capsid.

Separate analysis of the effects of each of the non-synonymous mutations located in the capsid proteins on both freezing–thawing resistance and replicative capacity of Qβ revealed interesting insights (Figure 5). Specifically, mutations G1494A, U1665C and G2223A were associated with a slight increase in freezing–thawing resistance, while mutation C2001U led to a decrease in resistance. Mutations G1312A and A1930G had no apparent effect. Regarding replicative capacity, this apparently was increased by mutations G1312A, C2001U and G2223A, whereas the remaining mutations showed no significant impact. The presence of mutation C2001U in line L1 is intriguing, since this mutation decreases the resistance to freezing and thawing. This mutation likely persists as a low-intensity polymorphism due to its beneficial effect on viral replicative capacity. Moreover, when mutation C2001U co-occurred with other mutations, such as in clone L1C1, no adverse effects on freezing–thawing resistance were observed.

The limited resistance to freezing and thawing observed in single mutants generated via site-directed mutagenesis again suggests that a combination of mutations within the same genome is what confers the resistance phenotype. To investigate this idea, we isolated several biological clones, sequenced their genomes, and evaluated their resistance after three F/T cycles. Among the four clones isolated from line L1, three exhibited higher resistance levels than the ancestor and the single mutants. However, their resistance was inferior to that of the complex population from which they came. A similar trend was observed for clones from line L2. These results are reminiscent of early studies that showed that individual biological clones displayed lower average fitness values than the entire population from which they had been isolated [61]. Later studies also demonstrated that cooperative interactions in RNA virus populations can enhance overall fitness beyond that of individual mutants, typically through replicative cooperation [62,63,64,65,66,67]. However, in this case, cooperation occurs among non-replicating mutants, posing a challenging question. One possibility to explain this kind of cooperation is the formation of virion aggregates, either with themselves or with the cellular debris produced after the lysis of infected bacteria. The formation of these aggregates could be favored by the presence of particular mutations, some of which might not be detectable in the consensus sequence determined by Sanger methodology, but could be in a high-throughput sequencing analysis. Although this possibility is purely speculative, it is worth noting that the association of certain viruses with particulate matter may increase their resistance in the external environment [68]. Also, previous work by our group identified a mutation (T222N) in the A1 protein of the Qβ capsid that appears to increase interaction with cellular debris [59,60]. These types of interactions could be favored in heterogeneous populations and be less relevant in clonal viruses. In contrast to what it is observed in lines L1 and L2, clones isolated from line L3 displayed resistance levels closer to the population, suggesting a more uniform behavior of mutants in this line. Further investigation is needed to uncover the specific interactions driving cooperative behavior among mutants that are not replicating and their role in enhancing overall viral fitness under freezing–thawing stress.

The observation that three independent lines, facing the same environmental challenge, employed different adaptive pathways underscores the presence of diverse mutational routes leading to similar phenotypic outcomes. This contrasts with previous experiments in our group, where we investigated Qβ resistance to heat shock at 60 °C in the extracellular medium [31]. In that scenario, a single mutation in the A1 protein of the capsid was sufficient to enhance resistance. Interestingly, a similar experiment conducted with heat shocks at 52 °C revealed a greater diversity of adaptive pathways [69], suggesting that the intensity of selective pressures can influence the degree of convergence in adaptive responses between lines. In our study, the variability in adaptive pathways under freezing–thawing stress indicates the presence of multiple mutational solutions to the challenge. Had the virus been subjected to a greater number of F/T cycles between replications, the heightened selective pressure might have resulted in fewer beneficial mutations and increased convergence in adaptive responses across lines.

Our results show that even mesophilic viruses can develop strategies to adapt to extracellular stresses like freezing and thawing, provided they have the opportunity to replicate after exposure to the negative condition. In the absence of that opportunity, viral extinction becomes a likely outcome. Such extinctions could be prevalent in ecosystems experiencing drastic environmental changes and/or where the availability of susceptible hosts remains low for extended periods. This underscores the significance of studies elucidating viral adaptation to environmental stressors, as they provide crucial insights into ecosystem dynamics and the regulatory role of viruses within them.

An aspect not addressed in this article is the relevance of adaptive strategies in disease-causing viruses. Host jumps, the emergence of variants resistant to antibodies or antiviral drugs, are but further consequences of the high evolutionary capacity of viruses, particularly those with RNA genomes. During severe epidemics, measures such as reducing interpersonal contacts may be implemented to reduce transmission. However, this can lead to viruses staying longer in the environment, favoring those with greater stability outside the cell for subsequent infections. This serves as yet another example of the importance of understanding how extracellular selective pressures shape viral evolution.

## 5. Conclusions

Bacteriophage Qβ can increase its resistance to the damage caused by the exposure to freezing and thawing cycles, as long as it has the ability to replicate between cycles, thus allowing the generation of mutants with adaptive potential to this condition.

The increase of resistance to freezing and thawing does not have a cost in Qβ replicative capacity.

Mutations observed in Qβ evolved lines were predominantly located in capsid proteins. Non-synonymous mutations possibly alter capsid structure, potentially increasing its resistance, while synonymous mutations likely influence viral RNA structure, modifying internal capsid pressure.

The limited convergence found in parallel-evolved viral lines indicates that there are multiple adaptive pathways that can lead to similar phenotypes.

Clones isolated from evolved lines exhibit resistance levels lower than the entire population, indicating potential cooperation among mutants.

## Figures and Tables

**Table 1:** Normalized survival rates for the virus lines and clones indicated.

Virus Line or Clones	Normalized Survival Rate ^1^	Virus Line or Clones	Normalized Survival Rate ^1^	Virus Line or Clones	Normalized Survival Rate ^1^
L1	19.3	L2	21.0	L3	6.0
L1C1	1.0	L2C1	7.4	L3C1	2.3
L1C2	8.2	L2C2	8.1	L3C2	3.1
L1C3	9.4	L2C3	5.5	L3C3	7.1
L1C4	5.3	L2C4	1.1	L3C4	5.3

^1^ The normalized survival rate was calculated with respect to the mean value of the survival rates obtained for the three clones isolated from the virus Qβ_Anc_ which are represented in Figure 6. This value was 0.03 ± 0.007.

## Data Availability

The datasets generated and/or analyzed during the current study are available from the corresponding author on reasonable request.

## Supplementary Materials

The following supporting information can be downloaded at: https://www.mdpi.com/article/10.3390/ijms25094863/s1.
