# Supplementary material for: Evolutionary Adaptation of an RNA Bacteriophage to Repeated Freezing and Thawing Cycles
Source: Int J Mol Sci. 2024 Apr 29;25(9):4863. doi: 10.3390/ijms25094863 (PMC11084849; doi:10.3390/ijms25094863)
Supplement: Supplementary file 1 [file ijms-25-04863-s001.zip › Table S1.pdf]

Table S1. Sequence of the complementary primers used to build the Qβ site-directed mutants

Mutant	Primer sequences[1]
$Q\beta_{G1312A}$	5′TATCTTTTTATTAACCCAACGC**A**TAAAGCGTTGAAACTTTGGGTC3′
	5′GACCCAAAGTTTCAACGCTTTA**T**GCGTTGGGTTAATAAAAAGATA3′
$Q\beta_{G1494A}$	5′GGAGAAGCGTGTTACC**A**TTTCGGTATCTCAGCC3′
	5′GGCTGAGATACCGAAA**T**GGTAACACGCTTCTCC3′
$Q\beta_{U1665C}$	5′CCGATGAGGAACGAGCT**C**TTGTTCGTACAGAGCTT3′
	5′AAGCTCTGTACGAACAA**G**AGCTCGTTCCTCATCGG3′
$Q\beta_{A1930G}$	5′TAATGCTGTTGAACTCC**G**GCCTCGCGAATTTGATG3′
	5′CATCAAATTCGCGAGGC**C**GGAGTTCAACAGCATTA3′
$Q\beta_{C2001U}$	5′CGTGATTGGGATTCTCGG**T**TTAGTTATACCACGTTCC3′
	5′GGAACGTGGTATAACTAA**A**CCGAGAATCCCAATCACG3′
$Q\beta_{G2223A}$	5′TCACGCCGATGGCGTGATA**A**TTGGCTTTTGGC3′
	5′GCCAAAAGCCAA**T**TATCACGCCATCGGCGTGA3′

[1]The nucleotide shown in black corresponds to the mutated position to be introduced in the Qβ genome
