# Supplementary material for: Evolutionary Adaptation of an RNA Bacteriophage to Repeated Freezing and Thawing Cycles
Source: Int J Mol Sci. 2024 Apr 29;25(9):4863. doi: 10.3390/ijms25094863 (PMC11084849; doi:10.3390/ijms25094863)
Supplement: Supplementary file 1 [file ijms-25-04863-s001.zip › Table S2.pdf]

Table S2. Mutations present in the evolutionary lines indicated

Mutation[1]	Aa change	Protein	Evolutionary line[2]			
			L1	L2	L3	Control
U471C	Syn	A2	-	P	-	-
A1065G	Syn	A2	-	-	F	-
G1312A	V417I	A2	-	P	-	-
G1494A	V50I	CP	-	P	-	-
U1665C	F107L	CP	F	-	-	-
C1760U	Syn	A1	-	-	-	P
A1930G	Q195R	A1	-	P	F	F
C2001U	L219F	A1	P	-	-	-
C2201U	Syn	A1	-	-	-	P
G2223A	V293I	A1	-	P	-	P
G2468A	Syn	Replicase	-	-	P	-
G2798A	Syn	Replicase	-	P	-	-
U4001C	Syn	Replicase	F	-	-	-

[1]For each mutation the amino acid change and the protein where it is located are indicated. In case that the mutations is synonymous it is indicated with Syn. P denotes polymorphic mutations and F fixed mutations.

[2]The evolutionary lines are described in Materials and Methods (section "Evolution experiment") and in Figure 1.
