# Supplementary material for: Evolutionary Adaptation of an RNA Bacteriophage to Repeated Freezing and Thawing Cycles
Source: Int J Mol Sci. 2024 Apr 29;25(9):4863. doi: 10.3390/ijms25094863 (PMC11084849; doi:10.3390/ijms25094863)
Supplement: Supplementary file 1 [file ijms-25-04863-s001.zip › Table S3.pdf]

Table S3. Mutations present in the consensus sequences of the virus clones indicated

Virus clones[1]	Mutations[2]				
	Non-coding regions	A2	CP	A1	Replicase
$Q\beta_{Anc}(1)$					
$Q\beta_{Anc}(2)$					
$Q\beta_{Anc}(3)$					U2972C
L1C1			**U1665C (F107L)**	**C2001U (L219F)**	U4001C
L1C2		**G1312A (V417I)**	**U1665C (F107L)**, C1689U	**A2187C (S281G)**	**U2847C (Y165H)**, U4001C
L1C3		C420U	**U1665C (F107L)**		G3341A, U4001C
L1C4			**U1665C (F107L)**		U4001C
L2C1		U969C, **G1312A (V417I)**		**A1930G (Q195R)**	
L2C2		U192C	**G1494A (V50I)**	**A1930G (Q195R)**, **G2223A (V293I)**	G2798A
L2C3		**G1312A (V417I)**		**A1930G (Q195R)**	**U2740C (M129T)**
L2C4		U393C, C1101U		**C1953U (L203F)**, C2201U	
L3C1		A1065G		**A1930G (Q195R)**	G2468A, U2951C
L3C2		U354C, A1065G		**A1930G (Q195R)**	G2468A, **U2847C (Y165H)**
L3C3	A54G	A1065G		**A1930G (Q195R)**	G2468A, U3314C
L3C4		A258G, A1065G		**A1930G (Q195R)**	G2468A

[1]The virus clones indicated are described in the section "Isolation of biological clones" of Materials and Methods.

[2]For each mutation, the protein where it is located is indicated. Non synonymous mutations are shown in bold letters and the corresponding amino acid changes are indicated in brackets. Common mutations between clones and the populations from which they were isolated (see Table S2) are shown in blue.
